# Incidence of animal poisoning cases in the Czech Republic: current situation

**DOI:** 10.2478/v10102-009-0009-z

**Published:** 2009-06

**Authors:** Helena Modrá, Zdeňka Svobodová

**Affiliations:** Department of Veterinary Public Health and Toxicology, Faculty of Veterinary Hygiene and Ecology, University of Veterinary and Pharmaceutical Sciences Brno, Czech Republic

**Keywords:** veterinary toxicology, Czech Republic, animal poisoning

## Abstract

This article reports the most frequent cases of poisoning in farm animals, horses, cats, dogs, wild animals, fish and honey-bees in the Czech Republic. At present, there are fewer cases of acute poisoning caused by high doses of toxic substances but there are more and more cases of chronic poisoning as a consequence of environmental pollution.

## Introduction

Veterinary toxicology is one of very important branches of veterinary medicine. Veterinary toxicology is specific in that its scope encompasses not only the identification and characterization of toxic substances, their physical and chemical properties, toxicodynamics and toxicokinetics, but, at the same time, the monitoring of the effects of toxic substances on a broad spectrum of animal species, from fish and bees to mammals.

At present, there are fewer cases of acute poisoning caused by high doses of toxic substances but there are more and more cases of chronic poisoning as a consequence of environmental pollution. Diseases caused by chronic exposure to toxic substances are very often non-specific, and manifested as a consequence of the immune system weakening or by decreases in weight gain in economically important animal species. Moreover, the accumulation of exogenous substances in tissues of economically important animal species also affects the safety and quality of food of animal origin.

In the Czech Republic, there is no central registry for veterinary poisonings. Of a total of about 10 thousand inquiries per year received by the Toxicological Information Centre (TIS), about one per cent are inquiries regarding veterinary poisonings. Most of the inquiries are about cat and dog poisoning, inquiries about poisoning in other animals are rare. Animal poisoning cases are investigated at State Veterinary Institutes and at the Faculty of Veterinary and Pharmaceutical Sciences in Brno, fish poisonings at the Research Institute of Fish Culture and Hydrobiology of the University of South Bohemia, and honey-bee poisonings at the Bee Research Institute at Dol near Libčice. The register of side effects associated with the administration of veterinary drugs is maintained at the Institute for the State Control of Veterinary Biologicals and Medicaments.

## Poisoning in farm animals

This section includes data on poisoning and effects of toxic substances in farm-raised ruminants, pigs and poultry.

Cases of acute poisoning with manifested clinical symptoms in ruminants are relatively infrequent. They are usually due to a gross breach of regulations governing the handling of toxic substances, non-observance of technological procedures, etc. An example of a gross breach of handling regulations for toxic substances and waste materials is the DDT cattle poisoning case that happened in 2002 at the Karlovy Vary region (Svejkovský *et al*., 2003).

The probability of poisoning and subsequent damage in different species and categories of ruminants differs according to the animal management system and feeding used. For barn-kept animals, the greatest risk is posed by contaminated or poor-quality feedstuffs (mycotoxins, nitrates, poisonous plants), improper doses of substances used as feedstuff supplements for ruminants (urea, mineral substances) and also by incorrect doses of administered drugs. Grazing ruminants, on the other hand, are at the greatest risk from the ingestion of poisonous plants, from contaminated water and zootoxins.

In recent years, we have very often met with mycotoxosis in cattle herds. Mycotoxins (and trichothecenes in particular) are at present a serious economic and health problem especially in calves and high-producing dairy cows. Most frequently, mycotoxicoses produce no overt clinical symptoms but as a chronic problem mycotoxicoses have very serious effects on economic performance of herds.

Likewise in ruminants, mycotoxins are the most frequent cause of sublethal intoxications also in pigs. In the past, ionophor (monensin, salinomycin) poisoning was often diagnosed in pigs. Because their use in pigs was banned in 2006, such cases no longer occur. Cases of classical kitchen salt poisoning, on the other hand, are quite common according to state veterinary data.

The main cause of sublethal mycotoxin poisoning of pigs are trichotecenes, ochratoxin A, zearalenon, fumonisins and aflatoxin B_1_. Of trichotecen mycotoxins, deoxynivalenol (DON) is the most frequent cause of poisoning in practice. Pigs suffering deoxynivalenol intoxication refuse feed and, at higher doses, DON indices vomiting in pigs (for that reason DON is also called vomitoxin). Other trichotecens, diacetoxyscirpenol (DAS) and T-2 toxin, cause reduction in feed intake of feeder pigs, lower weight gains and reduced resistance against secondary bacterial and viral infections. Ochratoxin A induces porcine neuropathy, fumonisins cause porcine pulmonary oedema (PPE), aflatoxin B_1_ is strongly hepatotoxic. The pig is the most sensitive species to the effects of zearalenon. Zearalenon is a strong oestrogen, and it causes reproductive disorders in sows and boars.

Veterinary hygienic protection of poultry herds on farms significantly restricts contacts with their external environments. Thanks to that, intoxications tend to be rather rare, and are usually the consequence of technology or the human factor on the farm. Recently a number of botulism cases in broiler chicken fattening operations have been reported (Rachač, 2002). The possible primary source of neurotoxin produced by *Clostridium botulinum* was contaminated straw bedding. In birds, botulism is predominantly caused by type C botulotoxin. The intoxication is characterized by specific clinical symptoms, i.e. loss of ability to control muscles, the absence of defensive reactions and of vocal manifestations. Particularly noticeable is the neck paralysis (the so-called limberneck).

## Equine poisoning

In the past, the most frequent were cases of poisoning with plants horses ingested when grazing. Among them the clearly predominating was the so-called Žd'ár disease caused by ragwort (*Senecio jacobea*) and *S. erraticus*. Because pastures where ragwort used to grow have been dried up, that type of cases has all but disappeared. In grazing horses, photodynamic dermatitis is diagnosed from time to time. It is caused by the effect of photo-sensibilizing plants. In specific reported cases the plants were the angelica (*Angelica archangelica*) and Saint John's wort (*Hypericum perforatum* or *H. maculatum*).

At present, poisonings in horses are rare. In 1998, a case of poisoning with salinomycin, which is used as an anticoccidicum in poultry, was reported in horses. In that particular case, the owner confused feed mixes and gave the horses a poultry mix containing salinomycin (Svejkovský *et al*., 2004). In 2002 and 2007, two cases of botulinotoxin poisoning in horses were reported (Jahn *et al*., 2008). Horses are very sensitive to botulotoxin exposure. The cause of intoxication is feeds contaminated with type B botulotoxin. The risk of botulotoxin intoxication is particularly high on farms where haylage is used as feed in which *Clostridium botulinum* has been allowed to propagate under anaerobic conditions.

Sublethal effects of atropin have been described in horses treated for uveitis. Uveitis is treated by repeated administration of 1–2% atropin in the form of eye drops until mydriasis is produced. Atropin overdose will slow down the peristalsis of the horses' intestines within 2–3 days, and obstipation and, subsequently, colic will occur.

## Poisoning in cats and dogs

Acute poisoning in cats and dogs is most frequently caused by their owner's negligence and use of off label drugs, but even intentional poisoning cases are no exception. The most frequent causes of poisoning in dogs at present still include anticoagulation rodenticides, and pesticides carbofuran and metaldehyde. Anticoagulation rodenticides poisoning may happen when dogs accidentally ingest a rodenticide bait in households but because of their easy availability, anticoagulation rodenticides are also often abused for intentional poisoning. No exceptions are carbon oxide poisonings, when in most cases dogs and/or cats are victims together with their owners, and poisoning with ethylene glycol, whose sweet taste makes it attractive for animals. Poisoning with carbofuran, a pesticide belonging among carbamates, is always intentional and it occurs at hunting grounds where foxes or martens are the intended victims. Cat poisoning may sometimes occur as a result of an improper use of synthetic pyrethroids and paracetamol (Svobodová *et al*., 2008).

Every year, the most frequent inquiries of owners and veterinary surgeons relating poisoning, according to the Toxicological Information Centre, are about metaldehyde-based moluscocides in the case of dogs and about synthetic pyrethroids in the case of cats. Also frequent are inquiries about rodenticides.

## Poisoning in wild animals

Wild animals can be considered as suitable indicators of contamination of individual ecosystems, particularly with pesticidal preparations. In spite of the increased attention paid to environmental contamination with pesticides, the incidence rate of acute pesticide poisonings is relatively low. In practice, the death of wild animals due to acute poisoning is diagnosed relatively often. They are, however, mostly phytotoxicoses (poisoning of roe deer with oil rape), botulism in aquatic birds, and intentional carbofuran poisoning cases.

With the extension of oil rape acreages and the introduction of the so-called double-zero oil rape varieties (i.e. low glucosinolate varieties, which reduces bitter taste of oil rape and thus enhances its palatability for animals), rape is consumed in excessive quantities by roe deer particularly in the winter and early sprint periods. The acute form of the poisoning is manifested by digestion disorders – mainly tympany, subchronic form, caused by poisoning with S-methyl cystein sulfoxide, which causes haemolytic anaemia with subsequent neurological symptoms (loss of fear of humans, disorientation, etc.).

Botulism in aquatic birds occurs in eutrophic ponds and reservoirs where, under anaerobic conditions, botulotoxin is produced in decomposing organic matter containing *Clostridium botulinum* (Rachač, 1986). Botulism in aquatic birds is caused by type 3 botulotoxin. The main symptom is the paralysis of all muscles, and characteristic is the so-called limberneck.

Carbofuran (the active ingredient of the preparation Furadan) has been the most frequent cause of poisoning in wild predatory birds in the Czech Republic in the past 10 years. Illegal use of carbofuran used to be a widespread practice in vermin (foxes, martens, etc.) control. Carbofuran was added to various types of bait (dead calf, fish, etc). Lethal doses of carbofuran for birds are about 10 times smaller than for mammals. Because of its high toxicity for birds, the most frequent were the deaths of wild predatory birds. As of 13 December, 2008, carbofuran-based preparations were banned. We hope that this will help to significantly reduce the incidence of carbofuran poisonings in the future.

Feral pig poisoning cases occur only exceptionally when feral pigs happen to come across some poisons that might be, e.g. deposited at illegal dump sites (kitchen salt, nitrogen fertilizers).

## Poisoning in fish

Accidental killing of fish due to the contamination of the aquatic environment remains among the most frequent poisoning cases. Every year, about 260 to 300 of such accidents are diagnosed. The largest number of the cases, both worldwide and in the Czech Republic, is related to oil spills, which do not kill fish but cause sensory changes to fish muscle tissue.

The following is the list of 6 most frequent causes of fish poisoning of last decade:

**Table:** 

organic substances and ammonia	40%
organic substances	35%
ammonia including auto-intoxication	10%
change in water pH	7%
metals (Fe, Al and others)	3%
others (nitrites, chlorine, etc.)	5%

In the past decade, no pesticide poisonings have been recorded, while in the 1970–1990 period they were responsible for 6% of fish poisoning.

The number of unresolved cases of accidental fish deaths remains very high (about 50%). Fish poisoning diagnostic is very difficult and complicated because the fish death is often found only after a period of time, and samples of fish and water (particularly of running water) are often taken too late. In such cases, toxic substances are usually already washed away from the originally affected sites and are not demonstrated in the samples taken. Moreover, pathomorphological changes may be masked by the onset of postmortal changes (particularly in summer months of the year). One of the most important pieces of information for fish poisoning diagnostics is the result of in situ examination.

Present-day research in fish toxicology focuses primarily on the monitoring of aquatic environment contamination and of the effects of residues of various substances in sublethal concentrations on fish. In addition to chemical monitoring, biological monitoring of aquatic environment contamination is also given much attention. The results of biological - and in particular biochemical – monitoring reflect the effects of all the substances present in the aquatic environment at the given location. Biochemical markers indicate contamination with broader groups of pollutants, e.g. cytochrome P450, EROD, conjugation enzymes indicate contamination with organic pollutants, and metallothioneins indicate metal contaminations. Specific biochemical markers include, e.g. 1-hydroxypyrene determined in fish bile, which serves as an indication of the environmental contamination with PAHs.

Effects of the residua of various substances persisting in the aquatic environment, the most important of those being pesticides and medical drugs, are also monitored. From among pesticides, the most frequently found are residua of triazine herbicides (e.g. of atrazine, terbutryn and simazine). From among medicinal drugs, frequently found are residues of substances of oestrogenic nature, and also of triclosan, propiconazole, etc. Effects of these substances at sublethal concentrations are monitored mainly from the point of their relationship to reproductive capacity in fish (Svobodová *et al*., 2008).

## Honey-bee poisoning

Bees constitute a very important group of livestock that come into a close contact with both natural toxins and contaminants of the environment. From time immemorial, bees come into contact with natural toxicants from nectar, honeydew and plant pollen. In the central European setting, natural toxins represent no major danger for bees. Thus all bee poisoning cases on record are therefore traceable to human activity. An important characteristic of bees is their social way of life in colonies, where an essential role is played by the passing food to each other by regurgitation. In doing so they pass also exogenous substances the food may contain. For that reason the greatest damage is caused by toxicants with a long residual efficiency period because in that case, the entire bee colony may get contaminated. Nowadays, mass intoxications due to immissions caused by industrial processing of low-grade coal and minerals are a matter of the past. Bee poisoning cases reported included poisoning with hydroxymethylfurfural (HMF) produced when sugar is heated under acidic conditions, due to an overdose or an improper administration of drugs.

The most important role in bee toxicology, however, is played by pesticides, and, in particular insecticides and, in some cases, also herbicides (paraquat and diquat herbicides).

The largest number of mass poisonings of bees has been caused by the insecticide Regent WP 50 containing fipronyl as the active ingredient. Its residual efficiency period is 21 days. The use of the preparation on oil rape against the rape blossom beetle has been banned since 2006. Although other applications of the preparation are still permitted, the year 2006 was the first year in the history of pesticide use when no case of mass poisoning of bees was officially reported on the territory of the Czech Republic. However, the danger of bee poisoning with pesticides still exists. Bees are still at risk of poisoning with organophosphates, carbamates, pyrethroids, and, in particular, with the newly introduced neo-nicotinoid pesticides. These preparations serve as fungicides and are used to treat seed grain. In other countries, and especially in the USA, they cause heavy losses to bee colonies.
